# Effective training of nanopore callers for epigenetic marks with limited labelled data

**DOI:** 10.1098/rsob.230449

**Published:** 2024-06-12

**Authors:** Brian Yao, Chloe Hsu, Gal Goldner, Yael Michaeli, Yuval Ebenstein, Jennifer Listgarten

**Affiliations:** ^1^ Department of Electrical Engineering & Computer Sciences, University of California, Berkeley, CA 94720, USA; ^2^ Department of Chemical Physics, Tel Aviv University, Tel Aviv-Yafo, Israel; ^3^ Edmond J. Safra Center for Bioinformatics, Tel Aviv University, Tel Aviv-Yafo, Israel; ^4^ Center for Computational Biology, University of California, Berkeley, CA 94720, USA

**Keywords:** nanopore, epigenetics, supervised training, limited training data

## Abstract

Nanopore sequencing platforms combined with supervised machine learning (ML) have been effective at detecting base modifications in DNA such as 5-methylcytosine (5mC) and N6-methyladenine (6mA). These ML-based nanopore callers have typically been trained on data that span all modifications on all possible DNA k -mer backgrounds—a *complete* training dataset. However, as nanopore technology is pushed to more and more epigenetic modifications, such complete training data will not be feasible to obtain. Nanopore calling has historically been performed with hidden Markov models (HMMs) that cannot make successful calls for k -mer contexts not seen during training because of their independent emission distributions. However, deep neural networks (DNNs), which share parameters across contexts, are increasingly being used as callers, often outperforming their HMM cousins. It stands to reason that a DNN approach should be able to better generalize to unseen k -mer contexts. Indeed, herein we demonstrate that a common DNN approach (DeepSignal) outperforms a common HMM approach (Nanopolish) in the incomplete data setting. Furthermore, we propose a novel hybrid HMM–DNN approach, amortized-HMM, that outperforms both the pure HMM and DNN approaches on 5mC calling when the training data are incomplete. This type of approach is expected to be useful for calling other base modifications such as 5-hydroxymethylcytosine and for the simultaneous calling of different modifications, settings in which complete training data are not likely to be available.

## 1. Introduction

Nanopore sequencing is a third-generation technology for sequencing DNA and RNA that provides advantages over other technologies, such as its small size, long-read lengths and real-time, mobile sequencing capabilities [1–3]. Additionally, nanopores are increasingly being used to detect epigenetic modifications to DNA, particularly DNA methylation [4]. The nanopore device works by running an ionic current through nanometer-wide pores. As a DNA molecule passes through the pore, the current across the pore changes in a manner that is characteristic of the molecules in the pore, namely, the RNA/DNA sequence and its modifications. From measuring the current on known sequences and modifications, one can build up a supervised training dataset suitable for machine learning (ML) methods that are then able to transform future, unlabelled current signals to their corresponding sequence of bases and modifications [5].

Early studies demonstrated that nanopore sequencing could be used for the detection of epigenetic modifications in DNA by using distinct current levels produced when a modified base is in the pore [6,7]. These successes sparked the development of supervised ML methods for methylation calling on nanopore data [8–11]. The first methylation modification tackled by nanopore technology was 5-methylcytosine (5mC), a well-studied modification owing to its abundance in the human genome [12] and its links to a number of key biological processes such as ageing and cancer [13,14]. Indeed, nanopore-based methylation calling has been shown to be an effective tool in the classification of brain tumours [15]. More recently there have been efforts to tackle a different cytosine modification, 5-hydroxymethylcytosine (5hmC), which is common in mammalian brain tissue, accounting for 40% of modified cytosine in the central nervous system [16]. 5hmC content in brain cells increases with age, suggesting that it is linked to neurodevelopment [17]. Early results suggest that nanopore current may be sensitive to 5hmC [6]. However, calling 5hmC accurately in the presence of other modifications has not yet been conclusively achieved, largely because of the difficulty in obtaining sufficient labelled training data for the ML-based callers [18].

### 1.1. Generalization capabilities of nanopore callers

Although supervised ML methods are developed specifically for their ability to generalize to unseen examples, the notion of generalization for nanopore sequencing is nuanced. For example, one form of generalization for base calling is from one set of current observations for one k -mer, to slightly different current observations for that same k -mer arising from stochastic noise in the system. We call this *sensor generalization*, because the generalization required is owing only to sensor noise. Another form of generalization relevant to nanopore sequencing is *k -mer generalization*, wherein an ML-based caller must make accurate calls for *k*-mers for which it has never seen current observations. Analogous notions of generalization can be made for epigenetic calling, where, in addition to k -mers comprising the standard nucleotides, we also consider *modified k -mers* that include methylated bases. In this case, k -mer generalization refers to generalizing the combination of both bases and modifications.

When constructing a training dataset for *base* callers (i.e. to read DNA/RNA sequences), it is relatively easy, for values of k commonly used in practice (i.e. *k=6 *), to generate a *k -mer complete* dataset—one in which current observations associated with all possible k -mers are present. This can be achieved by taking, for example, a sample of human DNA of known sequence, amplifying the DNA and running it through the nanopore. Corresponding labels for training can be obtained by sequencing those same reads with an alternative sequencing platform at high enough coverage/confidence to be certain of the sequence. In summary, it suffices to require only sensor generalization for base calling because we can readily obtain a *k -mer complete* training dataset.

However, when it comes to constructing a training dataset for, say, a particular methylation modification, it can be more difficult to obtain a similarly comprehensive dataset. This difficulty arises from the burden of obtaining high-confidence labels for these modifications—that is, in obtaining the ground truth bases and modifications for sequences run through the nanopore. In previous studies of 5mC modifications, we could rely on either enzymatically methylated DNA [10,11] or the gold standard assay of bisulfite sequencing to obtain supervised labels [19,20]. However, as we move to other modifications, such as 5hmC, obtaining ground truth data at scale will be more difficult. For example, the current standard methods for single-base resolution calls of 5hmC, namely, T-assisted bisulfite sequencing and oxidative bisulfite sequencing (oxBS) [21] are expensive and low-throughput [22,23]; they also require high coverage to make high-confidence 5hmC calls (particularly oxBS) [21]. Additionally, beyond these sequencing challenges, the rarity of certain epigenetic modifications may also present a problem, as it may be the case that not all k -mers containing a given modification are represented in a specific genome, and synthesizing each one is not typically feasible. As the field progresses to the simultaneous calling of multiple types of epigenetic modifications, achieving a complete dataset with respect to all of the modifications and bases will become harder still [18]. Consequently, as nanopore sequencing technology is pushed to call more and more DNA modifications, we will require ML-based callers that are accurate even with relatively incomplete training data. In particular, the callers will require both k -mer and sensor generalization.

To further illustrate these difficulties, consider that for base calling, the nucleotide alphabet is of size four: {A, C, G, T}, whereas for a given methylation mark that can occur only on a cytosine, we expand the alphabet to size five: {A, C, G, T, M}. For pore models where k=6 , we go from $4^{6}=4096$ unique k -mers to $5^{6}=15\;625$ . Additionally, even if the pore contains only, say, six bases at a time, ML callers may be able to make use of larger contexts, such as nine, to improve calling [24]; this exacerbates the combinatorial explosion of possible k -mers ( $5^{9}=1\;953\;125$ ). Similarly, simultaneously calling of even two distinct modification types, such as 5mC and 5hmC, would dictate an alphabet of size six, corresponding to $6^{6}=46\;656$ unique k -mers. Herein, we restrict ourselves to k=6 and to calling a single modification (5mC), although the conclusions that emerge should be equally, if not more applicable to larger values of k and to situations in which there are multiple epigenetic modifications of interest.

Next, we describe the two main modelling approaches currently used for nanopore-based methylation calling, and discuss how each is able or not able to perform sensor and k -mer generalization. Then, we propose a new approach, which is a hybrid between the two existing approaches, and demonstrate its use in the limited training data regime. Note that in both of these existing modelling approaches, and in our own, the methylation calling assumes that base calling has already been performed, although the overall message of our work does not depend on this.

### 1.2. Hidden Markov model nanopore callers

Simpson *et al.* [10] developed the widely used Nanopolish, a hidden Markov model (HMM)-based approach to detecting 5mC in cytosine-guanine dinucleotide (CpG) contexts. The Nanopolish HMM assumes a different current distribution for each unique k -mer, including distinct distributions for modified versions of a k -mer. For example, a k -mer, CGAACG, that has a 5mC in the fifth position, denoted CGAAMG, has its own mean and variance of current distribution in Nanopolish, and MGAAMG in turn has its own and so forth. That is, every possible modification on top of any DNA background—a unique modified k -mer—has its current distribution modelled independently. The Markov transitions in the HMM ensure a coherence of calls as the DNA sequence moves through the pore. That is, if the HMM believes the last call in the sequence being pulled through the pore was a CAMGAT, then the next call in the sequence should be offset by a shift of one, AMGATX, for wildcard X. Because of the independent current distributions—called emission distributions in HMM parlance—for each modified k -mer, the HMM-based Nanopolish approach cannot accurately make calls for modified k -mers not seen in the training data.

### 1.3. Deep neural network nanopore callers

Recently, there has been a shift to using deep neural networks (DNN) for base [25–27] and methylation calling [19,20]. In particular, Ni *et al.* [20] created DeepSignal by employing a bidirectional recurrent neural network (RNN) with long short-term memory (LSTM) units to construct sequence-based features, jointly with a convolutional neural network to process the current. Liu [19] similarly used a bidirectional LSTM (biLSTM)–RNN in their DeepMod, also adding a secondary neural network to account for the correlation of modifications on nearby sites. More recently, Ahsan [28] improved on this architecture in DeepMod2 by allowing the model to incorporate information from further sequencing timesteps and additionally implementing a transformer model to be used in place of the original biLSTM layers. These DNN-based methods have shown improved performance over the HMM-based Nanopolish for 5mC calling [29]. Importantly, because these DNN approaches do not have parameters that are *a priori* independent for each modified k -mer, it stands to reason that they should perform better than HMM-based approaches in generalizing to new modified k -mers—that is perform better k -mer generalization. Although it has not previously been shown, we will demonstrate that this is indeed the case.

### 1.4. A novel hybrid hidden Markov model–deep neural network approach to methylation calling

We hypothesized that combining the two modelling approaches may provide better k -mer generalization yet, and therefore better robustness to incomplete training datasets. The HMM is inherently a low-capacity model, with relatively few parameters, while DNNs typically have order of magnitudes more parameters, require vast amounts of data to train and also potentially days to weeks of architecture search to find a useful model. Our approach, amortized-HMM, starts by training a Nanopolish HMM on the training data that is available, yielding an emission distribution for each modified k -mer in the training data. Next, from the trained HMM, we extract each modified k -mer and its corresponding learned emission distribution. With these pairs, we then train a feedforward deep neural network (FDNN) to learn a mapping from modified k -mer to HMM emission distribution parameters. Now, we have all the ingredients for our final model, which makes calls on new test examples as follows. We use the Nanopolish HMM, except that we augment any missing emission distributions with the one predicted by the FDNN. Thus, the FDNN is in effect imputing missing emission distributions for modified k -mers not in the training data. Thus, with our modelling strategy, information sharing between emission distributions is possible by way of the FDNN. Consequently, we say that we are amortizing the emission distributions, hence the name, amortized-HMM. Although one could use only the FDNN emission distribution parameters for all modified k -mers (including those present in the training data), we found that this did not perform as well (see §4 and the electronic supplementary material, figure S1). In addition to developing this hybrid model, we also developed a practical method for optimally choosing which modified k -mers to use for training in the k -mer incomplete setting (see §2.1).

Next, we present a series of experiments comparing and contrasting our proposed hybrid approach to pure DNN and HMM approaches, across a range of k -mer incompleteness settings. We show that for complete training data, the DNN is superior to both amortized-HMM and Nanopolish, but that as the training data become increasingly incomplete, our hybrid approach dominates both other approaches in performance.

## 2. Results

We focussed our empirical investigation on the problem of 5mC calling, for which several high-quality datasets exist, and for which existing callers have been developed with the intent of having approximately k -mer complete training data. This investigation serves as a proof-of-principle for harder tasks such as 5hmC calling or joint calling of 5mC and 5hmC and so forth, for which we could not conduct such experiments owing to the lack of complete training data motivating this very work. Our comparisons used existing datasets from naturally occurring 5mC modifications in human genomes [19,20]. In particular, we conducted two sets of experiments by training and evaluating Nanopolish, DeepSignal and amortized-HMM on nanopore datasets that had been obtained by running two different human genomes, HX1 [8] and NA12878 [30] through the nanopore. We obtained gold standard bisulfite 5mC labels for NA12878 from ENCODE (ENCFF835NTC) [31] and for HX1 by processing bisulfite sequencing data from the NCBI Sequence Read Archive (PRJNA301527) [8] using Bismark [32].

### 2.1. Construction of incomplete training datasets

From both of these datasets, we independently did the following. First, we constructed a range of k -mer incomplete training datasets in order to assess how different callers performed in different settings of incompleteness. To do so, we used a six-fold cross-validation experimental set-up. We began by splitting the sequencing reads into six folds. Each fold contained approximately k -mer complete data. To simulate differing amounts of incompleteness, say, 10%k -mer complete training data, we first carefully (see below) selected a set of modified k -mers corresponding to 10% of the total number of possible modified k -mers (roughly 250 modified k -mers for 10% ). Then, for a given held-out fold for testing, we effectively excluded modified k -mers that had not been selected from the five remaining folds (see §4 for details). We then trained each model on the combination of these k -mer incomplete folds, and evaluated them on the held-out test fold with no k -mer filtering (i.e. we always evaluate on a k -mer complete test set). We repeated this procedure six times, with each fold being held out for evaluation once.

As alluded to above, we developed our own approach for carefully selecting the modified k -mers used for training. In principle, for the above example, we could have simply chosen 250 of the training modified k -mers at random for our 10% complete dataset. However, this would not correspond to a real physical situation owing to the fact that a single methylated site in a genome would yield, when put through the nanopore, six modified k -mers (for k=6 ), all shifted from each other by one position—a structure that random sampling would not capture. To account for this physical constraint, we used a modified k -mer selection scheme whereby we enforced that all six modified k -mers for one modified site were simultaneously included (or not) in the training data. Consequently, our modified k -mer incomplete training data corresponded to a realistic sequencing experiment. More specifically, our selection was achieved algorithmically by using an integer linear program (ILP), as detailed in §4. In this framework, one can also account for the distribution of frequencies of modified k -mers in the target genome, as we did herein. Note that each individual modified k -mer will generally appear many times in the training data (with distinct sensor readings), but the total number of unique k -mers is limited, dictated by the specified level of incompleteness.

**Figure 1.**
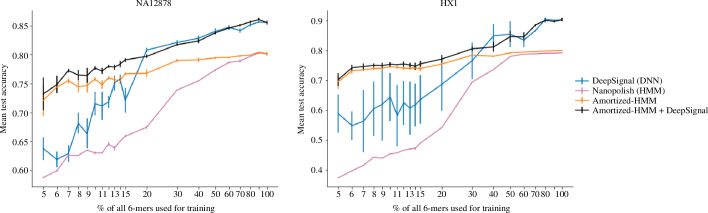
Performance of 5mC calling across different k -mer incompleteness regimes. Results averaged over six-fold cross-validation, with length of error bars equal to 1 s.d. across the folds. The k -mer complete case ( p=100 ) contains 2669 unique modified k -mers, whereas p=5 contains 133 . The units of test accuracy were per cent modified, meaning the best accuracy was 0, and the worst was 1.

We denote different levels of k -mer completeness by p . That is, p is the percentage of all possible unique modified k -mers that are present in the training data. A k -mer complete dataset has p=100 , while increasingly less complete datasets have increasingly smaller values of p —in principle down to p=0 , although in our experiments, we went only as low as p=5 , which corresponded to fewer than 150 unique modified k -mers in the training data. The 20 values of p used can be seen on the horizontal axis in figure 1.

We trained three different methylation callers, described in the previous section: Nanopolish (HMM), DeepSignal (DNN) and amortized-HMM, on each k -mer incomplete dataset. For the first two, we used code provided by the authors. No model selection or architecture search was performed for these methods because Nanopolish does not require it, and architecture search for DeepSignal had already been performed for the k -mer complete setting. For fairness of comparison of our approach to DeepSignal, we performed an architecture search for amortized-HMM only in the k -mer complete setting (see §4).

### 2.2. Accuracy of 5-methylcytosine calling across a range of k -mer incompleteness

On both datasets, the performances of Nanopolish and amortized-HMM were very similar for high values of p (figure 1). This is to be expected, since when p is close to 100 , amortized-HMM does not need to impute many emission distributions; rather it can use the Nanopolish emission distributions directly. Consistent with earlier results, we find that DeepSignal outperforms Nanopolish in the k -mer complete setting [20,29], and similarly outperforms amortized-HMM in that same setting, for similar reasons.

As the training data become increasingly incomplete (figure 1), amortized-HMM starts to systematically outperform Nanopolish because of its ability to impute missing emission probabilities corresponding to modified k -mers not in the training data.

Meanwhile, DeepSignal continued to hold an advantage over the other methods for p higher than around 20–30, the cross-over point for where amortized-HMM starts to outperform DeepSignal. We hypothesize that the diminished performance of DeepSignal with increasingly k -mer incomplete data arises from insufficiently informative data to warrant the high model capacity. Note that both DeepSignal and amortized-HMM had their architectures tuned only on the basis of k -mer complete training data, so as to make the comparison fair. However, it is possible that performing architecture search for each of the two approaches could have altered their relative performance. To investigate this hypothesis, we performed an architecture search only for DeepSignal, at p=10 —a regime of significant k -mer incompleteness and one where amortized-HMM substantially outperformed DeepSignal. Although the architecture search improved the performance of DeepSignal, amortized-HMM, critically with *no hyperparameter tuning*, always outperformed DeepSignal (electronic supplementary material, figure S1). These results suggest that the amortized-HMM approach is more robust to changes in training data completeness, mitigating the need to redo computationally intensive architecture searches for different deployment scenarios.

Although amortized-HMM performed best for our main regime of interest—low values of p —we considered whether combining amortized-HMM with DeepSignal could yield an all-around best performer. Therefore, we made another combined approach wherein we used DeepSignal to make predictions on modified k -mers that occurred in the training data, and amortized-HMM for the rest. Indeed, this combined approach yielded an overall best method (figure 1). In the following investigations, we do not include this approach because our intent there is to understand the behaviour of the individual models that it combines.

### 2.3. Decomposition into sensor and k -mer generalization

**Figure 2.**
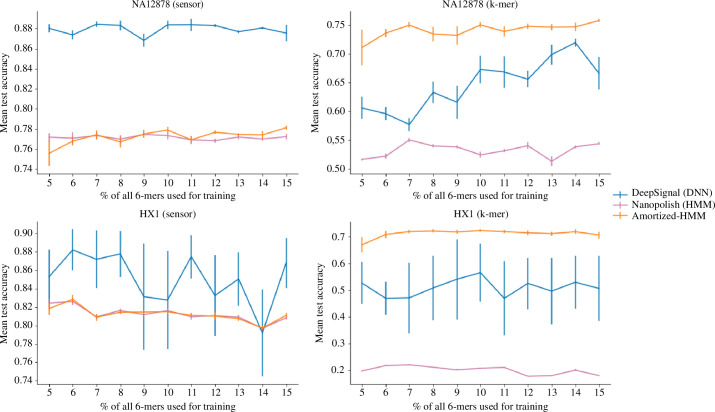
Sensor and k -mer generalization. Accuracy of the three approaches when the test set is broken down into modified k -mers observed at training time (sensor), and not observed (*k*-mer + sensor). General figure information is the same as in figure 1.

In order to better understand the source of each approach’s errors with respect to test modified k -mers that appeared in the training data (sensor generalization), and not ( k -mer generalization), we partitioned the test sets according to whether the modified k -mer had been seen in the training data or not (figure 2). Across both datasets, DeepSignal was the clear winner for pure sensor generalization (calling modified k -mers already present in the training data), while Nanopolish and amortized-HMM performed similarly to each other, and well below DeepSignal. On the other hand, when k -mer generalization was required, amortized-HMM consistently achieved the highest accuracy across all values of p , followed by DeepSignal, then Nanopolish. These results suggest that amortized-HMM better avoids overfitting on k -mer incomplete training data and consequently performs better on previously unseen, out-of-distribution modified k -mers.

### 2.4. Investigation of low- and high-novelty in k -mer generalization

**Figure 3.**
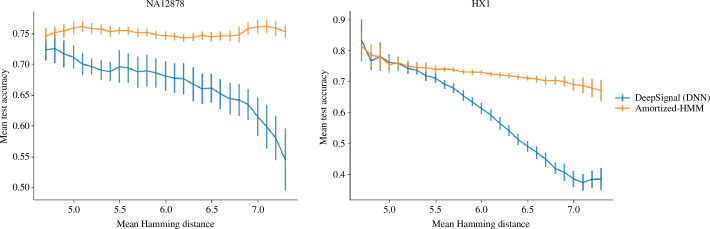
Breakdown into low- and high-novelty test set accuracy. Accuracy of DeepSignal and amortized-HMM on k -mers previously unobserved during training at varying levels of k -mer novelty. Results are averaged over values of p between 5 and 15 from figure 2. General figure information is the same as in figure 1. Hamming distance was computed on 11-mers containing consecutive 6-mers as described in §2.1 and §4.

In the previous section, we treated any modified k -mer not appearing in the training data in the same way, regardless of how similar it may have been to one of the training modified k -mers. However, we hypothesized that it may be the case that this similarity plays a role in how well the various models do. Therefore, we refer to test set-only modified k -mers that were less similar to any training examples as *high-novelty* test cases, and those that are more similar (but still different) as *low-novelty*. Similarity was defined by the Hamming distance of the one-hot encoded modified k -mers, and similarity to the training data were obtained by averaging this quantity over all unique modified k -mers in the training data. Using this definition of similarity for novelty, we compared the different approaches over all values of p between 5 and 15 appearing in figure 2, averaged over p (figure 3). Although amortized-HMM performed similarly to DeepSignal for low-novelty k -mers, it was far more accurate than DeepSignal for high-novelty k -mers. This difference in performance appears to underpin the success of amortized-HMM over DeepSignal in k -mer generalization.

## 3. Discussion

We investigated the effectiveness of three nanopore 5mC calling approaches—two existing common ones, and our newly developed method—in generalizing to modified k -mers not seen at training time—what we refer to as the task of k -mer generalization. Although the DNN-based DeepSignal performed best with k -mer complete training data, as the training data became increasingly less and less complete, our newly proposed hybrid approach that combines HMMs and neural networks, amortized-HMM, dominated in methylation calling accuracy.

Although we focussed our evaluation on 5mC detection, in practice, 5mC calling does not require effective k -mer incomplete modelling, as there already exists high k -mer coverage nanopore sequencing labelled datasets for 5mC. This setting was used as a proof-of-principle towards making progress on modifications for which obtaining such a dataset is not so straightforward. In particular, we are working to improve calling for 5hmC and naturally occurring combinations of cytosine modifications.

We note that both the existing callers investigated herein, and our own approach made calls having assumed that base calling has already been performed. In practice, it could be useful to combine the two tasks together for further improvements, as can be done in Guppy, the base caller developed by Oxford Nanopore Technologies (ONT; [18]).

## 4. Methods

### 4.1. Datasets

We trained and validated models for 5mC calling on two published Nanopore datasets. Jain [29] sequenced the human genome NA12878 at 30× coverage using the ONT R9.4 pore chemistry, and Liu [19] sequenced the HX1 genome, also at 30× coverage and using the R9.4 pore chemistry. In both cases, sequencing was performed on native DNA molecules containing native modifications; this is in contrast to other works where methylation was synthetically introduced in polymerase chain reaction-amplified samples using enzymes such as M.SssI methyltransferase [10,11]. In addition to raw current data, these datasets included base calling results obtained from running ONT-trained base callers: Guppy v.2.3.8 for the NA12878 dataset and Albacore v.2.3.1 for the HX1 dataset. To obtain supervisory 5mC labels for training our caller, we followed Liu [19] and Ni [20]. That is, first, we obtained gold standard bisulfite sequencing 5mC labels: for NA12878, these were downloaded directly from ENCODE (ENCFF835NTC) [31]; for HX1, we downloaded bisulfite sequencing results from the NCBI Sequence Read archive (PRJNA301527) [19], and pre-processed them using Bismark [32]. Then we filtered to keep only CpG sites that were: (i) covered by at least five reads; and (ii) were consistently called as methylated or unmethylated across every read. This process yielded a set of CpG sites for which we could confidently assign binary (methylated or unmethylated) labels for training and evaluation purposes.

### 4.2. *k*-mer selection for simulating incomplete training data

The motivation of this study is that one is unlikely to have k -mer complete training data for many epigenetic nanopore-calling problems of interest. This problem arises from, for example, limitations in experimental time and cost, or the modification of interest occurring infrequently in the genome in a given genomic context. However, since the datasets considered in this work are, by intent, k -mer complete, we simulated incomplete datasets by selecting only certain k -mers to retain. The goal of this selection procedure was to: (i) mimic the physical constraints of pulling a sequence through a pore (described soon); and (ii) select a diverse, representative set for training so as to most clearly see the effects of incomplete training data. Note that we focussed only on limiting the number of unique *modified* k -mers present in the training data, since one can assume that training data for k -mers with no modifications can be procured easily. For simplicity of language, we refer to the modified k -mers as k -mers in this section. Throughout all of our experiments, the number of sites in the pore at any one time, k , was 6 , although our procedure can be readily applied for other values of k . Also note that we consider only 5mC in CpG contexts, which is representative of mammalian methylation [33].

Let S denote the set of unique 6 -mers that contain a methylated CpG site. As described in the main text, p denotes the percentage of all possible modified k -mers that our incomplete dataset should contain. For example, if p=100 , then we retain all 6 -mers in S . For p<100 , requiring that we limit our training data to $\left\lfloor \frac{p\,\left|S\right|}{100}\right\rfloor$ unique 6 -mers, we might consider selecting them at random. However, such an approach would yield a training set not obtainable from an actual nanopore experiment because it is not accounting for the fact that as a sequence gets pulled through the pore, the corresponding 6 -mers that arise are overlapping. Augmenting the nucleotide alphabet with M to represent a methylated cite, consider the 11-mer, GATTTMGCAAC, centred on one methylated site. This 11 -mer comprises six overlapping 6 -mers: in order, GATTTM, ATTTMG, TTTMGC, TTMGCA, TMGCAA, and MGCAAC, that would arise from pulling it through the pore. In our selection scheme, we, therefore, constrain ourselves to select at the 11 -mer level, which directly implies a selection of 6 -mers at once. We refer to this as a *coherent* k -mer selection scheme, because it adheres to the physical reality of pulling a DNA strand through the pore. The random k -mer selection scheme is not coherent.

To ensure a coherent k -mer selection scheme, we formulate our k -mer selection problem as an ILP—an optimization of a linear function subject to a set of linear constraints over integer variables. In our setting, the linear function takes as input the presence/absence of each 11 -mer ( $y_{i}\in \left\{0,1\right\}$ for i=1,…,m , where m denotes the total number of possible unique 11 -mers centred on a methylated site), and returns the frequency-weighted count of the 11 -mers to be retained in the data (equation 4.1). The frequency weights, $w_{i}\in \left[0,1\right]$ (with $1=\sum_{i}w_{i}$ ), for any given 11 -mer, are specified by the user. As detailed above, the ith -mer comprises a set of 6 -mers, denoted $V_{i}$ . The presence or absence of an individual 6 -mer, s , is denoted by $x_{s}\in \left\{0,1\right\}$ . The two constraints of our ILP are that the number of selected unique 6 -mers is less than our budget, $B=\left\lfloor \frac{p\,\left|S\right|}{100}\right\rfloor$ (equation 4.2), and the other ensures k -mer coherence (equation 4.3). Altogether, our ILP is given by:


(4.1) $${\text{max}}_{\left\{y_{i}\right\}}\sum_{i=1}^{m}w_{i}y_{i},$$

(4.2) $$s.t.\left(\sum_{s\in S}x_{s}\right)\leq B,$$

(4.3) $$0\leq -ky_{i}+\left(\sum_{s\in V_{i}}x_{s}\right)\leq k-1\;\forall_{i}\in \{1,...,m\}.$$

Equation (4.3) may be more easily understood by noting its equivalence to the k -way logical AND constraint, $y_{i}={⋀}_{j=1}^{k}x_{i_{j}}$ , where ⋀ denotes a logical AND operator. The frequency weights could correspond to prevalence in a human genome or could be set to uniform. In our experiments, we set the weights to be the frequencies of the methylated 11 -mers observed in each labelled training dataset. In a more realistic scenario where methylation labels are not available, a reasonable proxy is to use the frequency of the unmodified 11 -mers; we found this also worked well (data not shown).

After solving the ILP with a standard solver (Gurobi [34]), we obtain values of *y*_*i*_, and correspondingly, *x*_*i*_, which dictates the 6 -mers we should keep in our training data. Had we instead tried to use a random selection scheme, and post hoc enforced coherence by removing 6 -mers that violated coherence, we would have ended up with dramatically fewer methylated sites in the training data (electronic supplementary material, figure S2).

We note that in a practical setting in which one cannot obtain k -mer complete data, one can also use this selection scheme to design a training dataset that will give optimal modified k -mer coverage subject to a fixed sequencing budget, e.g. by determining what modified k -mers to synthesize while also potentially factoring in k -mer frequency in a genome of interest. However, our intent here was simply to simulate incomplete training data from our k -mer complete 5mC data in order to investigate the effect of increasingly less complete data on different ML-based calling approaches. Our original intent had been to use a random selection scheme, but we then realized it was not coherent.

### 4.3. Training with Nanopolish and DeepSignal

We used publicly available software for Nanopolish [10] with default settings. In this software, first the current time series in each Nanopore read is segmented into events, which are then aligned to a reference genome. Then, each k -mer is associated with a list of events. Second, these lists of events are then used to update the emission distribution parameters for each k -mer in the HMM. This process is repeated from the alignment step for five iterations. In order to test how Nanopolish performs given k -mer incomplete training data, we only included events aligned to modified k -mers selected by our above procedure, so as to enforce that emission distribution parameters are not learned for excluded modified k -mers.

We used software publicly provided by DeepSignal with default settings. For a given CpG site in the reference genome and a read covering the site, DeepSignal extracts a feature vector containing nucleotide sequence information, current summary statistics and raw current values corresponding to a window centred on the CpG dinucleotide. For evaluating DeepSignal in the k -mer incomplete training data setting, we remove all feature vectors corresponding to modified k -mers excluded by our selection procedure from the training dataset.

There are two notable side effects of our k -mer selection downsampling. First, since we are only concerned with removing methylated sites from the data, our procedure naturally introduces a significant class imbalance in the training data between methylated and unmethylated k -mers, since all unmethylated sites remained. Since the HMM is class-imbalance agnostic, we did not need to account for these issues for Nanopolish. However, for DeepSignal, we found it was needed. Consequently, to mitigate this bias, we correspondingly randomly downsampled the negative (unmethylated) class at random, such that the proportion of each class was the same. Second, as we decreased, p , the number of allowed unique k -mers decreased, and correspondingly, the amount of training data. In order to keep the size of the training data (which can comprise multiple reads for the same k -mer), we randomly downsampled our training data so that we always had the same number of reads corresponding to the smallest value of p in our experiments, p=5 . Altogether, this left us for DeepSignal with around 1 million CpG sites for NA12878, and 4 million for HX1, for any value of p .

### 4.4. Amortized-hidden Markov model

Our new approach, amortized-HMM, extends the HMM method for methylation calling through the use of a DNN. From Nanpolish HMM training, we obtain emission distribution parameters (a scalar mean and a scalar variance) for k -mers observed at high enough frequency in the training data. We take these as labels for an FDNN that learns a mapping from k -mers to emission distribution parameters. Next, we impute any missing (i.e. those that Nanopolish left to the default) emission distributions with our FDNN, before using Nanopolish to make calls. In using only FDNN-based emission distributions by overriding all Nanopolish ones, performance was diminished (electronic supplementary material, figure S3).

Our FDNN requires us to featurize each k -mer given as input to the FDNN, which we do as follows for each k -mer. We use a one-hot encoding with the alphabet {A, C, G, T, M}, where M denotes a methylated site, with the other letters denoting nucleotides, yielding 30 binary features. We also one-hot encode each dinucleotide formed from adjacent positions, yielding 105 binary features. Finally, we use a binary encoding of whether the position contains a C/M, or does not, yielding six more binary features. The motivation for including this last feature is that M is closely tied to C by way of being a modified cytosine, so we hypothesized that they may have similar effects on the nanopore current. In total, the feature vector was of length 141 . Note that we additionally experimented with one-hot encoding trinucleotides from consecutive triplets in the k -mer, but this did not improve performance.

Rather than the more common losses used for training neural networks, we used a symmetrized Kullback-Leibler (KL) divergence loss to train our FDNN because the labels were themselves parameters of a distribution. Had we used say mean squared error, the numerical difference between the FDNN outputs and the labels would probably not meaningfully reflect the difference in distributions. In particular, we let $P∼N\,\left(\mu ,\sigma^{2}\right)$ be a Gaussian random variable with μ and $\sigma^{2}$ estimated from Nanopolish (i.e. our supervisory labels), and let $\hat{P}∼N\,\left(\hat{\mu },{\hat{\sigma }}^{2}\right)$ be a Gaussian variable with $\hat{\mu }$ , and ${\hat{\sigma }}^{2}$ predicted by the amortized-HMM. The symmetrized KL-divergence between these is defined as $f(P,\hat{P})=D_{KL}(P\;\|\;\hat{P})+D_{KL}(\hat{P}\;\|\;P)$ , whose components can be computed in closed form by: $D_{KL}(P\;\|\;\hat{P})=\log\frac{\hat{\sigma }}{\sigma }+\frac{\sigma^{2}+(\mu -\hat{\mu }{)}^{2}}{2{\hat{\sigma }}^{2}}-\frac{1}{2}$ and $D_{KL}(\hat{P}\;\|\;P)=\log\frac{\sigma }{\hat{\sigma }}+\frac{{\hat{\sigma }}^{2}+(\hat{\mu }-\mu {)}^{2}}{2\sigma^{2}}-\frac{1}{2}$ .

Architecture search was performed for amortized-HMM only for the k -mer complete setting, with the same selected hyperparameters being used for all experiments irrespective of the value of p . We determined the number of hidden layers, d , and the size of each hidden unit, h , with an 80 %/ 20 % random split of the data. We performed a grid search over the hyperparameter values d∈{3,4,5,6} and h∈{16,32,64,128} .

### 4.5. DeepSignal hyperparameter search in a k -mer incomplete data regime

For an apples-to-apples comparison with amortized-HMM, and to reduce the substantial computational burden, almost all of our reported experiments used architecture searches only from the k -mer complete setting (i.e. the default architecture provided by DeepSignal). However, as noted in the main text, we did perform an architecture search for DeepSignal for a single salient value of p=10 , to see if this would allow it to outperform amortized-HMM (without doing this added architecture search). The value p=10 corresponds to a regime of low k -mer coverage and one where our approach substantially outperformed DeepSignal (figure 1). We used the NA12878 dataset for training and validation. Furthermore, we tuned the same network hyperparameters that were originally tuned by Ni *et al*. [20]: the length of the k -mer context, the number of BRNN layers and the number of inception layers. Despite doing so, DeepSignal did not outperform amortized-HMM (electronic supplementary material, figure S1).

## Data Availability

All datasets used throughout this study were obtained from publicly available sources. The NA12878 nanopore sequencing dataset was downloaded from [35]. The NA12878 bisulfite methylation reference was downloaded from ENCODE (ENCFF835NTC). The HX1 nanopore sequencing dataset was downloaded from SRA (PRJNA533926). The HX1 bisulfite sequencing dataset was downloaded from SRA (PRJNA301527). Code for amortized-HMM is available as a GitHub repository under the GPL license [36]. Supplementary material is available online [37].

## References

[1] Branton D *et al* . 2008 The potential and challenges of nanopore sequencing. Nat. Biotechnol. **26** , 1146–1153. (10.1038/nbt.1495) 18846088 PMC2683588

[2] Jain M , Olsen HE , Paten B , Akeson M . 2016 The Oxford Nanopore MinION: delivery of nanopore sequencing to the genomics community. Genome Biol. **17** , 239. (10.1186/s13059-016-1103-0) 27887629 PMC5124260

[3] Quick J *et al* . 2016 Real-time, portable genome sequencing for Ebola surveillance. Nature New Biol. **530** , 228–232. (10.1038/nature16996) PMC4817224 26840485

[4] Gouil Q , Keniry A . 2019 Latest techniques to study DNA methylation. Essays Biochem. **63** , 639–648. (10.1042/EBC20190027) 31755932 PMC6923321

[5] Rang FJ , Kloosterman WP , de Ridder J . 2018 From squiggle to basepair: computational approaches for improving nanopore sequencing read accuracy. Genome Biol. **19** , 90. (10.1186/s13059-018-1462-9) 30005597 PMC6045860

[6] Laszlo AH , Derrington IM , Brinkerhoff H , Langford KW , Nova IC , Samson JM , Bartlett JJ , Pavlenok M , Gundlach JH . 2013 Detection and mapping of 5-methylcytosine and 5-hydroxymethylcytosine with nanopore MspA. Proc. Natl Acad. Sci. USA **110** , 18904–18909. (10.1073/pnas.1310240110) 24167255 PMC3839702

[7] Schreiber J , Wescoe ZL , Abu-Shumays R , Vivian JT , Baatar B , Karplus K , Akeson M . 2013 Error rates for nanopore discrimination among cytosine, methylcytosine, and hydroxymethylcytosine along individual DNA strands. Proc. Natl Acad. Sci. USA **110** , 18910–18915. (10.1073/pnas.1310615110) 24167260 PMC3839712

[8] Liu Q , Georgieva DC , Egli D , Wang K . 2019 NanoMod: a computational tool to detect DNA modifications using nanopore long-read sequencing data. BMC Genomics **20** , 78. (10.1186/s12864-018-5372-8) 30712508 PMC6360650

[9] Rand AC , Jain M , Eizenga JM , Musselman-Brown A , Olsen HE , Akeson M , Paten B . 2017 Mapping DNA methylation with high-throughput nanopore sequencing. Nat. Methods **14** , 411–413. (10.1038/nmeth.4189) 28218897 PMC5704956

[10] Simpson JT , Workman RE , Zuzarte PC , David M , Dursi LJ , Timp W . 2017 Detecting DNA cytosine methylation using nanopore sequencing. Nat. Methods **14** , 407–410. (10.1038/nmeth.4184) 28218898

[11] Stoiber M , Quick J , Egan R , Eun Lee J , Celniker S , Neely RK , Loman N , Pennacchio LA , Brown J . 2017 De novo identification of DNA modifications enabled by genome-guided nanopore signal processing. bioRxiv. (10.1101/094672)

[12] Breiling A , Lyko F . 2015 Epigenetic regulatory functions of DNA modifications: 5-methylcytosine and beyond. Epigenetics Chromatin **8** , 24. (10.1186/s13072-015-0016-6) 26195987 PMC4507326

[13] Gonzalo S . 2010 Epigenetic alterations in aging. J. Appl. Physiol. (1985) **109** , 586–597. (10.1152/japplphysiol.00238.2010) 20448029 PMC2928596

[14] Horvath S , Raj K . 2018 DNA methylation-based biomarkers and the epigenetic clock theory of ageing. Nat. Rev. Genet. **19** , 371–384. (10.1038/s41576-018-0004-3) 29643443

[15] Kuschel LP *et al* . 2023 Robust methylation-based classification of brain tumours using nanopore sequencing. Neuropathol. Appl. Neurobiol. **49** , e12856. (10.1111/nan.12856) 36269599

[16] Loh Y-HE , Koemeter-Cox A , Finelli MJ , Shen L , Friedel RH , Zou H . 2017 Comprehensive mapping of 5-hydroxymethylcytosine epigenetic dynamics in axon regeneration. Epigenetics **12** , 77–92. 27918235 10.1080/15592294.2016.1264560 PMC5330438

[17] Szulwach KE *et al* . 2011 5-hmC-mediated epigenetic dynamics during postnatal neurodevelopment and aging. Nat. Neurosci. **14** , 1607–1616. (10.1038/nn.2959) 22037496 PMC3292193

[18] Amarasinghe SL , Su S , Dong X , Zappia L , Ritchie ME , Gouil Q . 2020 Opportunities and challenges in long-read sequencing data analysis. Genome Biol. **21** , 30. (10.1186/s13059-020-1935-5) 32033565 PMC7006217

[19] Liu Q , Fang L , Yu G , Wang D , Xiao CL , Wang K . 2019 Detection of DNA base modifications by deep recurrent neural network on Oxford Nanopore sequencing data. Nat. Commun. **10** , 2449. (10.1038/s41467-019-10168-2) 31164644 PMC6547721

[20] Ni P , Huang N , Zhang Z , Wang DP , Liang F , Miao Y , Xiao CL , Luo F , Wang J . 2019 DeepSignal: detecting DNA methylation state from nanopore sequencing reads using deep-learning. Bioinformatics **35** , 4586–4595. (10.1093/bioinformatics/btz276) 30994904

[21] He Y , Jang HS , Xing X , Li D , Vasek MJ , Dougherty JD , Wang T . 2020 DeepH&M: estimating single-CpG hydroxymethylation and methylation levels from enrichment and restriction enzyme sequencing methods. Sci. Adv. **6** , 27. (10.1126/sciadv.aba0521) PMC7458459 32937429

[22] Jurkowski TP . 2020 Technologies and applications for the assessment of 5-Hydroxymethylcytosine. In Epigenetics methods, vol. 18: Translational Epigenetics (ed. T Tollefsbol ), pp. 261–278. Cambridge, MA: Academic Press.

[23] Rivera CM , Ren B . 2013 Mapping human epigenomes. Cell **155** , 39–55. (10.1016/j.cell.2013.09.011) 24074860 PMC3838898

[24] Simpson JT . 2019 Nanopolish. See http://github.com/jts/nanopolish/tree/r10.

[25] Boža V , Brejová B , Vinař T . 2017 DeepNano: deep recurrent neural networks for base calling in MinION nanopore reads. PLoS ONE **12** , 1–13. (10.1371/journal.pone.0178751) PMC5459436 28582401

[26] Huang N , Nie F , Ni P , Luo F , Wang J . 2019 An attention-based neural network basecaller for Oxford Nanopore sequencing data. In 2019 IEEE Int. Conf. on Bioinformatics and Biomedicine (BIBM), San Diego, CA, USA, pp. 390–394. (10.1109/BIBM47256.2019.8983231)

[27] Wick RR , Judd LM , Holt KE . 2019 Performance of neural network basecalling tools for Oxford Nanopore sequencing. Genome Biol. **20** , 129. (10.1186/s13059-019-1727-y) 31234903 PMC6591954

[28] Ahsan MU , Gouru A , Chan J , Zhou W , Wang K . 2024 A signal processing and deep learning framework for methylation detection using Oxford Nanopore sequencing. Nat. Commun. **15** , 1448. (10.1038/s41467-024-45778-y) 38365920 PMC10873387

[29] Yuen ZWS , Srivastava A , Daniel R , McNevin D , Jack C , Eyras E . 2021 Systematic benchmarking of tools for CpG methylation detection from nanopore sequencing. Nat. Commun. **12** , 3438. (10.1038/s41467-021-23778-6) 34103501 PMC8187371

[30] Jain M *et al* . 2018 Nanopore sequencing and assembly of a human genome with ultra-long reads. Nat. Biotechnol. **36** , 338–345. (10.1038/nbt.4060) 29431738 PMC5889714

[31] Encode Project Consortium . 2012 An integrated encyclopedia of DNA elements in the human genome. Nature **489** , 57–74. (10.1038/nature11247) 22955616 PMC3439153

[32] Krueger F , Andrews SR . 2011 Bismark: a flexible aligner and methylation caller for Bisulfite-Seq applications. Bioinformatics **27** , 1571–1572. (10.1093/bioinformatics/btr167) 21493656 PMC3102221

[33] Luo C , Hajkova P , Ecker JR . 2018 Dynamic DNA methylation: in the right place at the right time. Science **361** , 1336–1340. (10.1126/science.aat6806) 30262495 PMC6197482

[34] LLC Gurobi Optimization . 2023 *Gurobi Optimizer reference manual*. See https://www.gurobi.com.

[35] Nanopore-Wgs-Consortium . 2019 Na12878. GitHub. See https://github.com/nanopore-wgs-consortium/NA12878.

[36] Yao B . 2021 Amortized-HMM. GitHub. See https://github.com/yao12310/AmortizedHMM.

[37] Yao B , Hsu C , Goldner G , Michaeli Y , Ebenstein Y , Listgarten J . 2024 Effective training of nanopore callers for epigenetic marks with limited labelled data. Figshare (10.6084/m9.figshare.c.7125275) PMC11286150 38862018

